# Myocardial steatosis and impaired energetics are independent predictors of regional contractile function in patients with severe aortic stenosis

**DOI:** 10.1186/1532-429X-15-S1-O27

**Published:** 2013-01-30

**Authors:** Masliza Mahmod, Sacha Bull, Joseph Suttie, Nikhil Pal, Cameron Holloway, Rajarshi Banerjee, Sairia Dass, Houman Ashrafian, Jurgen E Schneider, Saul Myerson, Jane M Francis, Theodoros Karamitsos, Stefan Neubauer

**Affiliations:** 1 Oxford Centre for Clinical Magnetic Resonance Research, Department of Cardiovascular Medicine, University of Oxford, Oxford, UK; 2 Department of Cardiovascular Medicine, University of Oxford, Oxford, UK

## Background

In aortic stenosis (AS), there is impairment of myocardial energetics and a substrate switch with a preference to glucose metabolism and downregulation of fatty acid utilization. Whether or not this results in cardiac steatosis is unknown. We hypothesized that cardiac steatosis is present in severe AS and this can be assessed by ^1^H magnetic resonance spectroscopy (MRS). We aimed to investigate myocardial lipid content in patients with severe AS and to examine the associations amongst steatosis, energetics and myocardial function.

## Methods

Thirty patients with severe AS (20 symptomatic, 10 asymptomatic) and 20 normal volunteers were enrolled. Inclusion criteria were peak aortic valve gradient >50 mmHg, no other significant valvular pathology, normal left ventricular systolic function, no obstructive coronary artery disease and blood pressure <160/90 mmHg. Myocardial lipid content and phosphocreatine/adenosine triphosphate (PCr/ATP) ratios were quantified using ^1^H and ^31^P MRS, respectively. Global left ventricular systolic function and mass were assessed using cine cardiac magnetic resonance imaging (CMR) and peak circumferential strain using CMR tagging.

## Results

Clinical characteristics and CMR parameters are presented in Table 1. All subjects were matched for age, gender and body mass index. Although global left ventricular systolic function was similar in all groups, peak systolic circumferential strain and PCr/ATP ratios were reduced in AS patients, with the greatest reductions seen in the symptomatic group. Myocardial lipid content was elevated in AS patients, with the greatest increase seen in the symptomatic group. Impaired peak systolic circumferential strain was associated with myocardial steatosis (R=0.44, p=0.002) and reduced PCr/ATP ratios (R=-0.54, p<0.001). Multivariable analysis indicated that myocardial steatosis (β=0.34, p=0.036) and reduced PCr/ATP ratio (β=0.53, p=0.004) were independent predictors of peak circumferential strain.

**Table 1:** Baseline characteristics and cardiac magnetic resonance findings of the study subjects

	Severe Aortic Stenosis		Normal (n=20)	P value
	
	Symptomatic (n=20)	Asymptomatic (n=10)		
Age (y)	67 ± 11	61 ± 18	62 ± 4	0.14
Male [n (%)]	13 (65)	8 (80)	10 (50)	0.23
Body mass index (kg/m^2^)	29 ± 4	28 ± 4	26 ± 4	0.09
Aortic valve area (cm^2^)	0.84 ± 0.15	0.87 ± 0.16	-	0.68
Peak aortic valve gradient (mmHg)	78 ± 17	66 ± 16	-	0.10
Left ventricular ejection fraction (%)	75 ± 6	73 ± 8	69 ± 4	0.10
Left ventricular mass index (g/m^2^)	98 ± 33^*^	91 ± 26^*^	53 ± 13	<0.001
Left ventricular wall thickness (mm)	16 ± 3^*^	15 ± 2^*^	9 ± 1	<0.001
Peak systolic circumferential strain (%)	-17.2 ± 2.5^*^	-18.6 ± 2.8^*^	-20.1 ± 1.6	0.002
PCr/ATP ratio	1.38 ± 0.17^†^	1.65 ± 0.26^*^	1.91 ± 0.29	<0.001
Myocardial lipid content (% of water)	0.85 ± 0.43^*^	0.74 ± 0.29^*^	0.44 ± 0.18	0.001

^*^p<0.05 vs normal, ^†^p<0.05 vs asymptomatic severe aortic stenosis and normal.

## Conclusions

This is the first study to demonstrate that myocardial steatosis, in addition to impaired energetics, occurs in severe AS regardless of symptoms, and is independently associated with impaired peak circumferential strain. This suggests that myocardial lipid assessment may serve as a useful adjunct to standard clinical information to determine the optimal timing for aortic valve replacement.

## Funding

None.

**Figure 1:**
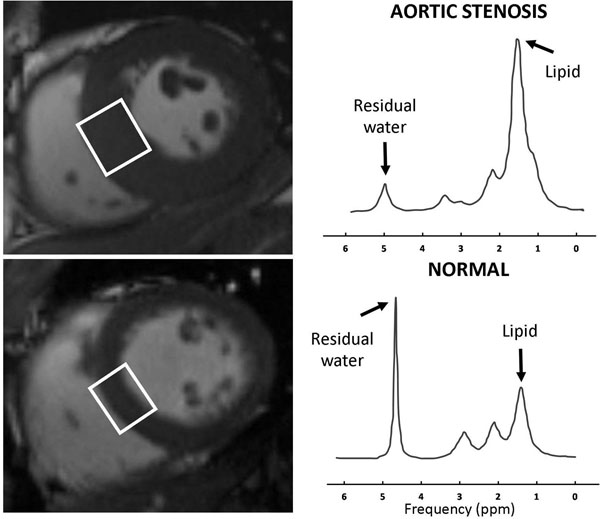
^1^H spectrum showing a prominent lipid peak in a patient with severe aortic stenosis (top) when compared with a normal subject (bottom). The voxel is placed in the mid-ventricular septum.

